# Target Motifs Affecting Natural Immunity by a Constitutive CRISPR-Cas System in *Escherichia coli*

**DOI:** 10.1371/journal.pone.0050797

**Published:** 2012-11-26

**Authors:** Cristóbal Almendros, Noemí M. Guzmán, César Díez-Villaseñor, Jesús García-Martínez, Francisco J. M. Mojica

**Affiliations:** Departamento de Fisiología, Genética y Microbiología, Facultad de Ciencias, Universidad de Alicante, Alicante, Spain; St. Petersburg Pasteur Institute, Russian Federation

## Abstract

Clustered Regularly Interspaced Short Palindromic Repeats (CRISPR) and CRISPR associated (*cas*) genes conform the CRISPR-Cas systems of various bacteria and archaea and produce degradation of invading nucleic acids containing sequences (protospacers) that are complementary to repeat intervening spacers. It has been demonstrated that the base sequence identity of a protospacer with the cognate spacer and the presence of a protospacer adjacent motif (PAM) influence CRISPR-mediated interference efficiency. By using an original transformation assay with plasmids targeted by a resident spacer here we show that natural CRISPR-mediated immunity against invading DNA occurs in wild type *Escherichia coli*. Unexpectedly, the strongest activity is observed with protospacer adjoining nucleotides (interference motifs) that differ from the PAM both in sequence and location. Hence, our results document for the first time native CRISPR activity in *E. coli* and demonstrate that positions next to the PAM in invading DNA influence their recognition and degradation by these prokaryotic immune systems.

## Introduction

Arrays of regularly spaced DNA repeats are present in 85% of sequenced archaea and about 50% of bacteria (CRISPRdb at http://crispr.u-psud.fr/crispr/, [1]). Even though the repeat sequence may vary to a great extent among arrays [2], the regular distance between repetitions grant their recognition as members of a family [3] at present known with the acronym CRISPR (Clustered Regularly Interspaced Short Palindromic Repeats; [4]). At least some of the repeat intervening sequences (spacers) are acquired from identical DNA fragments (protospacers) in bacteriophages and plasmids [5]–[9]. Functionally related to CRISPR, and usually in close proximity to them, are the *cas* (CRISPR associated) genes [4], [10], [11], altogether conforming the CRISPR-Cas systems. Diverse systems, currently classified into three main types (I, II and III) each including several subtypes, are distinguished mainly based on the presence of particular signature *cas* genes [12]. Increasing numbers of biochemical and genetic studies indicate that CRISPR-Cas provides adaptive immunity against molecules carrying protospacers. Indeed, specific Cas endonucleases cleave protospacers after base pairing complementary spacers carried in small CRISPR RNA (crRNA) molecules (for recent reviews on the CRISPR-Cas systems see [13], [14]). Additional sequence elements adjacent to repeat-spacer arrays (i.e. the leader) or to protospacers (i.e. the protospacer adjacent motif denoted PAM) participate in this activity. Notably, the leader contains promoters for the transcription of the adjacent CRISPR array [15]–[20] and is required for insertion of repeat-spacer units at the leader proximal edge of the repeat cassette [9]. PAMs are short (2–5 nt) signatures located next to one end of the protospacers. The sequence and location of the PAM, relative to that of the corresponding spacer in the CRISPR array, is conserved for systems with similar repeats (belonging to the same CRISPR type according to Kunin et al. [2]) but both may vary among CRISPR types [21]–[27]. PAMs are required for efficient interference by at least some CRISPR-Cas systems [5], [22], [27]–[29] and their occurrence strongly suggests that they are recognized by the acquisition machinery during the selection of spacer precursors [25].

Two CRISPR-Cas systems, pertaining to subtypes I-E and I-F [12], also known as Ecoli and Ypest respectively [10], have been identified in *E.coli* strains [30]. With two exceptions, represented by *E. coli* strain B7A and *Shigella* sp. D9 that contain both systems, either none or just one apparently functional system (with repeats and a complete set of associated *cas* genes) is retained. Subtype I-E is prevalent within the species and has been the subject of multiple studies (see recent publications [6], [8], [9], [31]). In contrast, subtype I-F is almost exclusively present (same two aforementioned exceptions) in a few members of the phylogenetic group B2 (4 out of 15 B2 strains of the ECOR collection; [30]) and functional studies on this system have only been performed in *Pseudomonas aeruginosa* [32]–[37].

The observation that most *E. coli* isolates harbour either I-E or I-F suggests that they can replace one to each other [30]. Indeed, they are strikingly similar structural and mechanistically. First, they pertain to the same main type (i.e. Type I), defined by characteristic processing and interference mechanistic details as well as the presence of *cas3* gene, which is fused to *cas2* in the case of I-F subtype [12]. Their *cas1* genes are more closely related to each other than to homologs in any other subtype [10], [12]. Apart from Cas2, Cas3 and Cas1 occurring in the two *E. coli* systems, the remaining Cas proteins do not show evident sequence homology [12], yet they constitute interference complexes (named Cascade and Csy-complex for I-E and I-F systems respectively) similar at the structure and topology level [31], [35], [38], that appear to be functionally analogous [39]. Furthermore, although I-E and I-F repeats pertain to distinct sequence types [2], both are partially palindromic [2] and have the shortest repeat periodicities (60 and 61 respectively) among known CRISPR [1], [10].

In this work we analyzed interference by the CRISPR-Cas I-F of *E. coli* LF82. This system is made of two arrays of CRISPR-4 repeats [2], accordingly referred to as CRISPR4.1 and CRISPR4.2 arrays respectively [30], separated by six *cas* genes (namely *cas1, cas2/cas3 fusion, csy1, csy2, csy3 and cas6f*). Putative leader sequences have been identified adjoining each array [30]. Here we show that, in contrast to the subtype I-E, which is silent under normal laboratory growth [17]–[19], [40], the Cas I-F genes are constitutively expressed and produce interference against target DNA in native conditions. The previously predicted PAM motif for CRISPR-4 repeats [25] is also observed in *E. coli*, but the most efficient interference motif (sequence in the PAM region causing interference) differs from that signature, showing a one-nucleotide displacement from the protospacer.

## Results

### Identification of the PAM Associated with the CRISPR-Cas I-F System of *E. coli*

**Figure 1:**
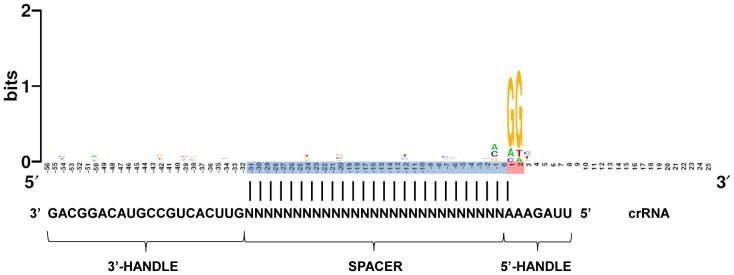
WebLogo generated by the alignment of protospacer regions of *E. coli* CRISPR-4 spacers. Protospacer and PAM positions are shaded in blue and red respectively. A crRNA molecule with an undefined spacer (N nucleotides) and the surrounding CRISPR sequences (5′ and 3′ handles) is drawn to illustrate the orientation of the PAM region with respect to the crRNA (aligned with adenine nucleotides at the 3′ end of the 5′ handle) when the spacer anneals to the cognate protospacer during target recognition.

In a previous study [25], the alignment of regions containing protospacers associated with CRISPR-4 repeats from *Pseudomonas aeruginosa*, *Yersinia pestis* and *Shewanella* spp. revealed the conservation of the PAM signature GG adjacent to the end of the protospacers that becomes leader proximal in the corresponding CRISPR array. Now we performed a similar analysis of regions containing protospacers of 36 CRISPR-4 spacers from *E. coli* strains (Figure S1). Their alignment confirmed the previously observed PAM also located towards the leader side (Figure 1). As shown in Figure 1, positions are numbered starting from the PAM-proximal end of the protospacer and increasing towards the 3′ end (i.e. 5′ protospacer-G_1_G_2_ 3′). In addition to the conservation of G_1_G_2_, exclusion of guanine at position 3 and thymine at position -1 was perceptible. However, the latter was dismissed as no nucleotide preference was evidenced by a further alignment of over 130 CRISPR-4 spacers for which similar sequences outside CRISPR loci were not found (Figure S2).

### The Cas I-F genes of *E. coli* are Expressed Under Normal Laboratory Growth Conditions

**Figure 2:**
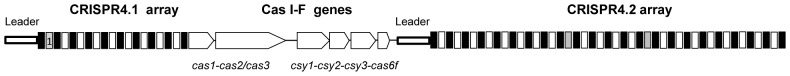
Graphical representation of the CRISPR-Cas I-F locus of *E. coli* strain LF82. Spacers with and without identified protospacers as well as repeats are represented as gray, white and black rectangles respectively. Spacer #1 is labeled. Cas genes are shown as boxes pointing towards the direction of transcription.

**Figure 3:**
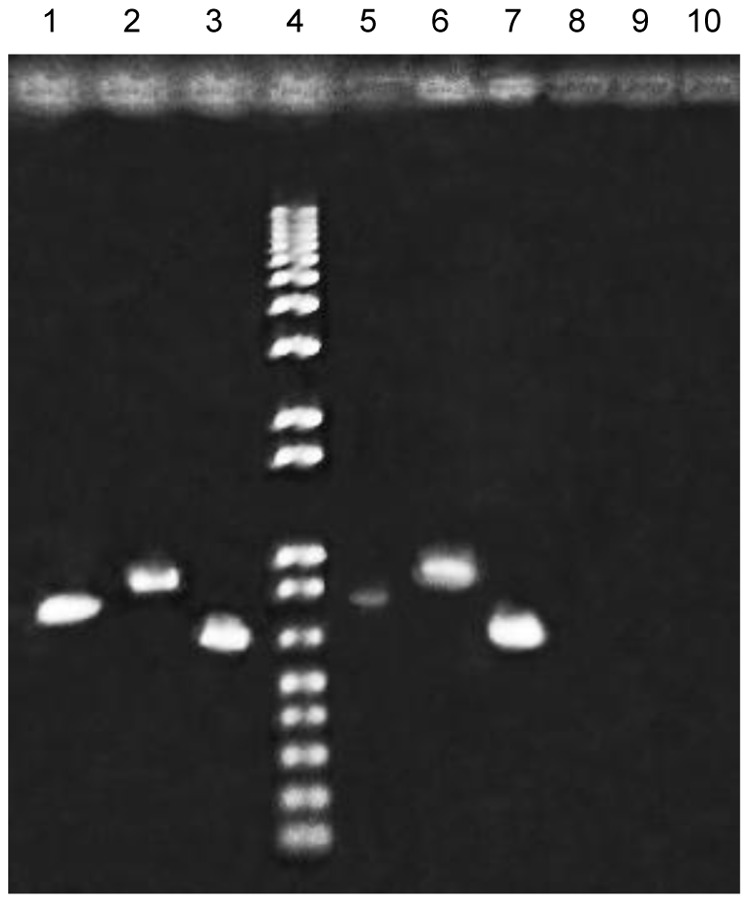
Expression of *cas1* and *csy1* genes revealed by RT-PCR. Agarose gel electrophoresis of PCR products obtained using as template total DNA (lanes 1 to 3), cDNA (lanes 5 to 7) or RNA (lanes 8 to 10) of LF82 strain grown in LB medium at logarithmic phase (results of samples from stationary phase cultures were similar and are not shown). In addition to *cas*1 (lanes 1, 5 and 8) and *csy1* (lanes 2, 6 and 9) the highly expressed *tufB* transcript (lanes 3, 7 and 10) was probed as a control of DNA contamination in RNA samples. A molecular weight marker is included (lane 4) for fragment size estimation.

While an array with two repeats is the only reminiscence of the CRISPR-Cas I-E system present in other *E. coli* strains, LF82 carries a typical CRISPR-Cas subtype I-F system [41] and its CRISPR4.1 and CRISPR4.2 arrays contain 9 and 22 spacers respectively (Figure 2). We searched by BLASTN sequences in non-CRISPR loci with over 90% identity to these spacers and found two matches within plasmids of the species and one in an enterophage (Figure 2 and Figure S1), suggesting that the CRISPR-Cas of LF82 could act as an immune system. In order to determine whether this system might be active under normal laboratory growth conditions, reverse transcription PCR (RT-PCR) experiments were performed with RNA purified from LF82 cultures grown in LB medium at logarithmic and stationary growth phase. The distance between open reading frames (ORFs) of the *cas* genes and the fact that all they have the same direction of transcription (see Figure 2) strongly suggests that they are organized into two transcription units (*cas2/cas3* and *csy1* ORFs are separated by 330 bp), the first one including *cas1* and *cas2/cas3* (ORFs separated by 3 bp) and the second spanning from *csy1* to *cas6f* (these ORFs are either overlapped or separated by less than 11 bp). Moreover, the proteins encoded by *csy1*, *csy2*, *csy3* and *cas6f* form a functional Csy-complex [16], [35], [39] and it has been reported for the analogous Cascade (Cas complex for antiviral defense) in the subtype I-E system that the corresponding genes form part of an operon [17]–[19]. Firstly, the possibility that the six *cas* genes were co-transcribed was dismissed as no product was obtained by total RNA reverse transcription followed by PCR amplification with primers targeting *cas2/cas3* and *csy1* (data not shown). Subsequently, the first gene of each transcription unit (*cas1* and *csy1*) was used as target for primers in PCR reactions of cDNA samples extracted from logarithmic and stationary phase cultures, obtaining amplification in all cases (Figure 3 and data not shown), hence demonstrating their expression in the conditions assayed.

### The CRISPR-Cas I-F System of *E. coli* Produces Interference Against Target Plasmids

**Figure 4:**
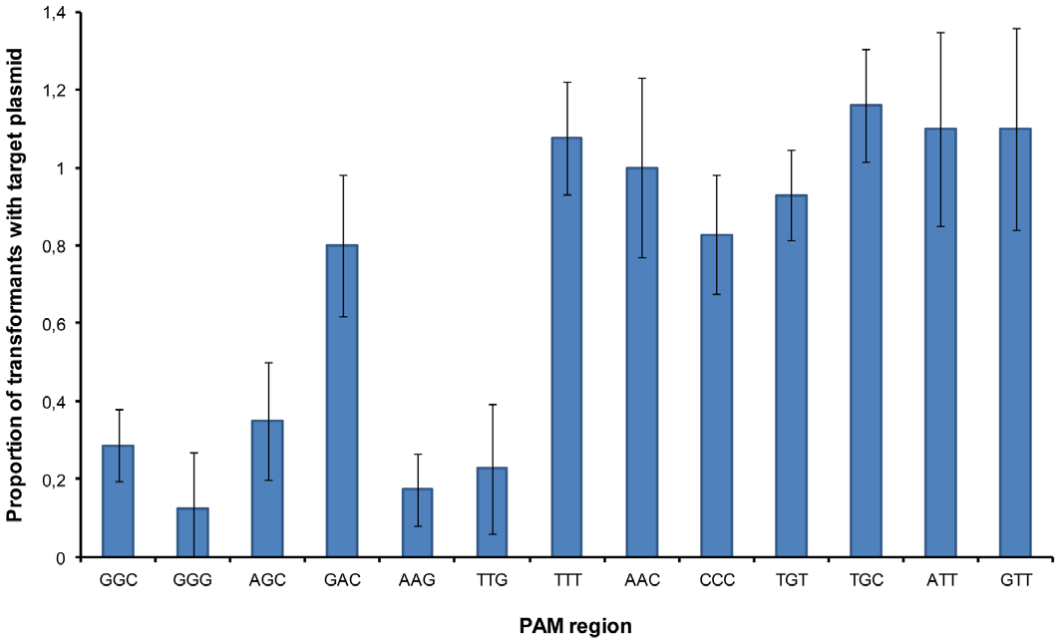
Histogram showing the results of transformation competition assays. Data correspond to the proportion of transformants carrying a target plasmid (containing protospacer #1) to transformant colonies carrying pCR2.1 (see material and methods for details). Target plasmids differ in sequence at positions 1 to 3 of the PAM region (the three nucleotides are indicated under the bars in that order). The mean average from three independent competition experiments for each targeted plasmid is shown with its standard deviation. Values significantly below 1 imply CRISPR interference.

Expression of the *cas* genes strongly suggests that the CRISPR-Cas I-F of LF82 is active, prompting us to investigate interference by this system. First, we tested natural interference by the first spacer after the leader of the CRISPR4.1 array of LF82, hereinafter referred to as spacer #1 (see Figure 2). LF82 cells were subjected to transformation with mixtures composed of an equivalent concentration of two plasmids that differ in the presence or absence of protospacer #1, a sequence identical to spacer #1. The proportion of transformants carrying the targeted plasmid for each experiment is an indication of the interference activity driven by spacer #1: strongest activity implies a lower proportion of transformants with the target plasmid (see material and methods). Initial transformation experiments were performed with plasmid pCR2.1, which has no sequence matching LF82 spacers, and pCAR-GGC, the latter carrying the tri-nucleotide GGC at the 3′ end of the protospacer #1. Interference with this PAM was made evident as, on average, 22% of transformants (p<0.01) carried pCAR-GGC (see Figure 4). Hence, the CRISPR-Cas I-F system of *E. coli* LF82 is naturally active against target plasmids.

### Identification of Nucleotides at the PAM Affecting Interference

Once CRISPR activity against protospacers with the predicted PAM was confirmed, the identification of nucleotides at the PAM region required for such interference, defining the interference motif, was addressed. With this aim, we performed competition assays with mixtures containing pCR2.1 vector and a derived plasmid (target plasmid) containing protospacer #1. Each transformation mixture differed in the sequence at positions 1 to 3 with respect to the protospacer in the target plasmid. Position 3 was included in the analysis as guanine was apparently excluded at this location and hence could form part of an extended PAM; i.e GGH (see Figure 1). Average data of three independent experiments for each plasmid pair are shown in Figure 4. Significance values corresponding to interference-deemed results were in all cases lower than 0.01. Strikingly, in addition to GGC and GGG, interference was observed with AGC, implying that G_2_ is enough for protospacer targeting. In contrast, the presence of guanine at position 1 had a limited effect on transformation balance (see GAC at Figure 4). Unexpectedly, G_3_ did also hold interference even in the absence of the canonical PAM G_1_G_2_ (see AAG and TTG) and moreover, the lower percentages of transformants carrying the target plasmids were invariably found when this third G was present (GGG, AAG, TTG). It is worth noting that the effect of G at positions 2 and 3 was cumulative (compare GGG with AGC and AAG). Taken together, these results show that at least one G either at position 2 or 3 is required for interference, the third G being the most effective. Strikingly, despite the high conservation of G_1_ in the CRISPR-4 PAM, this is neither sufficient nor necessary for such activity, a conclusion further supported by the equivalent transformation rates observed with GGC and AGC.

The potential influence of base pairing between the PAM region and the crRNA was also addressed. It has been demonstrated for the CRISPR-Cas type III system of *Staphylococcus epidermidis* that base pairing with the protospacer region beyond the spacer sequence of a crRNA prevents interference [42]. As illustrated in Figure 1, PAM positions 1 to 3 will stand face to face to adenine nucleotides at the 5′ handle of crRNA molecules after spacer-protospacer hybridization during target recognition in the interference stage. Although no interference was observed when the tri-nucleotide TTT was located adjoining the protospacer, a result that is compatible with interference prevention by base pairing, it could also be explained by the absence of guanine residues at the PAM. In the same context, it might be possible that the interference observed when guanines are present at the PAM is exclusively due to the absence of base pairing (they face adenine residues). However, this possibility is ruled out as neither AAC nor CCC produces interference confirming that guanines are specifically required at the PAM region as predicted from the results discussed before. Yet, prevention of interference by base pairing may be possible as interference held by G_2_ is abolished when the surrounding nucleotides are complementary to the corresponding positions in the crRNA (compare TGT and TGC to AGC, GGC and GGG in Figure 4). In contrast, and concurring with the strong interference held by G_3_, base paring at positions 1 and 2 did not affect interference when this G is present (compare AAG with TTG). Further studies will be required to assess the implication of crRNA-PAM annealing on interference.

## Discussion

In this work we show for the first time the occurrence of CRISPR-Cas activity against foreign DNA in wild-type *E. coli.* Natural immunity by a resident spacer of the CRISPR-Cas I-F system of LF82 strain was evidenced using an original assay (the competition test) based on the transformation efficiency of a target plasmid compared to that of the non-targeted parental vector. Instead of the widely utilized method that relays on independent transformation experiments for each plasmid, in the competition test cells are subjected to transformation with a mixture of both plasmids, each with equivalent concentration, purity and topology. Electroporation variables can greatly differ among experiments and therefore the rate of transformation. The competition test circumvents the influence of electroporation variables across independent experiments as the number of transformants carrying the targeted plasmid is normalized respect to an internal control (the non-targeted vector) instead of with data from a separate assay. In addition, competition tests using the same internal control are comparable. This advantage is especially relevant when small differences between plasmids are expected as an elevated experimental error may conceal subtle variations. In this context, the plasmids used for the competition assay are high copy number (pUC origin) and as a consequence, the screening of transformant colonies will reveal interference only when it happens soon after the target plasmid gets into the cell (before it reaches a high copy number). Our assay provides statistically significant data at differences in interference between targeted plasmids as low as two-fold.

It is expected that the selection of spacer precursors by the recognition of an adjoining motif (i.e. the PAM) has a functional meaning [25], [29], [31], [43] and nucleotides at the PAM have been shown to be important for interference [27], [28], [43]. However, in contrast with the PAM, the interference motif of at least some systems admits certain flexibility as shown here for a CRISPR-Cas I-F as well as in previous studies with other subtypes [6], [8], [22], [23], [29], [31], [43]. Although we have not tested the 64 possible tri-nucleotides at the PAM region, our results obtained with selected combinations clearly demonstrate that just one guanine residue, either at position 2 or 3, is sufficient to hold interference, but G at position 1 is not required, defining interference motifs that are shifted one position from the predicted PAM.

Previous works inferred PAM positions important for interference based on the selection of mutants that escape CRISPR interference. In this case, variations in the PAM that increase interference efficiency cannot be detected. In contrast, our study explores alternative nucleotides at PAM positions revealing both strong and weak interference motifs. Further, we show that positions outside the protospacer and PAM influence interference.

Two aspects related to the identity of the interference motifs versus PAM are intriguing: the dispensability of G_1_ and the implication of G_3_. Perhaps the role of G_1_ justifying its presence in the PAM is just to avoid base pairing with adenine in the crRNA, hence allowing interference by G_2_. The fact that interference is held by G_3_ in itself and, moreover, that the strongest interference is observed when this guanine is present is enigmatic because it is excluded of the PAM region of CRISPR-4 protospacers of *E. coli* (only 3 out of 36 protospacers carry it) and, notably, also of *Shewanella* spp. [25]. This apparent paradox could be justified from a biological perspective: a strong interference might be less advantageous for the cell than a relaxed one that would provide the opportunity for harmless and eventually beneficial foreign DNA to be acquired and maintained. In this vein, we defined in a previous work [25] the PAMs of 6 CRISPR repeat types. CRISPR-4 was the only one associated to the PAM G_1_G_2_, but CRISPR-1 and CRISPR-7 repeats, which like CRISPR-4 were linked to Type I Cas genes conforming subtypes I-B and I-A CRISPR-Cas systems respectively, had N_1_G_2_G_3_. It is evocative that the most active interference motif of CRISPR-4 differs from its PAM and coincides with the one of closely related repeats [2], [12]. This might reflect an evolutionary alternative that could be of particular benefit for the homogenous group (mostly within phylogenetic group B2) of *E. coli* strains containing CRISPR-Cas I-F systems. From a biochemical point of view, the fact that G_3_ is involved in interference may be just a consequence of the same Cas protein(s) being responsible for the detection of guanine in the PAM region during acquisition (G_1_G_2_) and interference (G_2_G_3_), both motifs being slightly displaced (one position) with respect to the protospacer, possibly as consequence of a slight displacement of the involved site of that protein when forming part of distinct nucleic acid-protein complexes.

The first report documenting interference activity by a CRISPR-Cas I-F system has been recently published for *Pseudomonas aeruginosa* (strain PA14) [36]. The *cas* gene content and layout of CRISPR-Cas I-F loci in *P. aeruginosa* PA14 and *E. coli* LF82 (hereinafter referred to as PaeIF and EcoIF systems respectively) are alike [32], [41]. Moreover, the amino acid identity percentage between Cas proteins of both systems (from about 40% to 65%) concurs with the phylogeny of the species [44]. Yet, the consensus CRISPR sequences are very similar (26/28 nt identity) [32], [41]. Such relatedness between both systems anticipates further mechanistic and functional analogy. Cady and collaborators [36] have now experimentally confirmed by spacer acquisition assays that sequences adjacent to the predicted PAM (GG) are selected as spacer precursors of this system and reported interference when this motif was present. Furthermore, in good agreement with our data, of three spacers conferring protection against phage infection which interference efficiency was estimated in *P. aeruginosa*, the target of the spacer showing the strongest interference adjoins G_1_G_2_G_3_. However, while our competition assays did not reveal a substantial effect of G to A substitutions at position 1, interference-evading phages were obtained during infection of *P. aeruginosa* with phages targeted by a PaeIF spacer where this nucleotide replacement was the only change observed at the protospacer region. This observation may suggest that, in contrast to EcoIF, G_1_ is essential for interference by the PaeIF system. Nevertheless, one evading phage lacking mutations in the target gene was also detected in this set of experiments, implying that a different cause is responsible for this resistance phenotype. That could also apply to the G_1_ to A_1_ change. A systematic analysis akin to the one we have employed in this work (i.e. independent of selection) would be required to confirm the requirement of G_1_ for interference by the PaeIF system.

In conclusion, the PAM of the naturally active I-F system of *E. coli* differs from the interference motifs. The presence of just one particular nucleotide in the PAM sustains immunity and adjacent positions affect its activity. These results could apply to other CRISPR-Cas systems, explaining why different targets with the same PAM show varied susceptibility.

## Materials and Methods

### *E. coli* Strains and Plasmids

*E. coli* strain LF82 used as a host for competition tests belongs to the phylogenetic group B2 [41] and contains a complete CRISPR-Cas I-F system.

Plasmids used in this work are described in Table S1. pCR2.1 (Invitrogen) is a high-copy-number cloning vector that confers resistance to ampicillin and kanamycin. pCR2.1 derivative plasmids carrying protospacer #1 and diverse adjacent sequences were constructed by ligation of PCR fragments, obtained with partially complementary oligonucleotide pairs, to the 3′-T overhangs of the linearized vector as supplied by the manufacturer (see Tables S1 and S2). PCR reactions were performed using Taq DNA polymerase (Roche) in a Mastercycler Gradient thermal cycler (Eppendorf, Wesseling-Berzdorf, Germany). Ligation of DNA fragments was performed with T4 Ligase (Roche) following the recommendations of the manufacturer and restriction enzymes were purchased from Fermentas.

### Plasmid DNA Purification and Quality Analysis

Plasmids were purified with the High Pure Plasmid Isolation Kit (Roche) following the manufacturer’s instructions. The DNA concentration and purity of samples was estimated with a Nanodrop ND-1000 (Nanodrop Technologies) and plasmid topology was analyzed by UV visualization of samples in EtBr stained agarose gels after electrophoresis in 1×TAE buffer.

### Transformation Procedure

Transformations were carried out by electroporation (2.45 KV, 25 µF, 200Ω) using an Electroporator 2510 (Eppendorf). Electrocompetent cells were prepared following the procedure described by Shi et al. [45]. Transformant colonies of pCR2.1 and derived plasmids were selected on LB agar plates containing 100 µg/ml ampicillin.

### Definition of the CRISPR-4 Protospacer Adjacent Motif (PAM) of *E. coli*

For the identification of the *E. coli* CRISPR-4 protospacer adjacent motif (PAM), regions of non-CRISPR loci containing sequences with over 90% identity to spacers of CRISPR-Cas I-F systems of *E. coli* strains were searched with the BLASTN program [46] run against the nr/nt database at the NCBI Website (http://blast.ncbi.nlm.nih.gov/Blast.cgi). When protospacers of different origin where found for a single spacer, the sequence with higher identity was selected for the analysis. Spacers were detected in *E. coli* sequences available through the coliBASE Website (http://www.xbase.ac.uk/colibase/) and in GenBank, using the CRISPR Finder application at http://crispr.u-psud.fr/ [1]. The DNA strands carrying the protospacer nucleotides complementary to the corresponding spacer sequence in the crRNA were aligned using the WebLogo application at http://weblogo.berkeley.edu/logo.cgi/to obtain sequence logos [47]. The ends of the protospacers were used as reference for alignments and no gaps were introduced.

### Competition Test Used for the Detection of Interference Activity

Interference by the native spacer #1 of *E. coli* strain LF82 was explored by competition tests. Briefly, in each experiment, LF82 cells were electroporated with plasmid mixtures (extracted from that strain) composed of the pCR2.1 vector and a derived construct carrying a sequence identical to spacer #1 (target plasmid). The DNA concentration, purity and proportion of the three topological forms (i.e. supercoiled, relaxed open circle and full-length linear) of each plasmid in each mixture was equivalent. Interference activity was estimated as the proportion of transformants carrying the target plasmid respect to those carrying pCR2.1 established by PCR screening. In the absence of interference, 50% of colonies harbouring either plasmid will be expected. But if spacer #1 produces interference with the protospacer carrier, a lesser percentage of cells transformed with this plasmid would be obtained, lower as interference activity increases. For each plasmid pair, three independent electroporation experiments, using different plasmid preparations and stocks of freshly prepared electrocompetent cells, were carried out. Twenty colonies were randomly selected from each experiment for PCR amplification. PCR reactions were performed under standard conditions using primers T7 (5′ GTAATACGACTCACTATAGGGC 3′) and M13 (5′ GGAAACAGCTATGACCATG 3′).

### Reverse Transcription Polymerase Chain Reaction Analysis (RT-PCR)

Total RNA was isolated from *E.coli* LF82 cells using the Trizol reagent (Invitrogen) as indicated by the manufacturer. About 200 ng of total RNA were retrotranscribed with hexameric random primers and SuperScript III retrotranscriptase provided in the SuperScript III kit (Invitrogen), according to the supplied protocol. cDNA was PCR amplified using primers for *cas1*, *csy1* and *tufB* genes (see Table S2). RT-PCR products were analyzed by UV visualization of EtBr stained agarose gels.

### Sequencing

Plasmid constructions were verified by sequencing with the Big Dye Terminator Cycle Sequencing kit in an ABI PRISM 310 DNA Sequencer following the manufacturer’s instructions (Servicios Técnicos de Invetigación, Universidad de Alicante, Spain).

### Statistical Analysis

Statistical analyses (Anova, Kruskal-Wallis and U Mann-Whitney tests) were calculated using SPSS software version 17.0 (SPSS 111 Inc., Chicago, IL, USA). A p-value less than 0.05 was considered as significant.

## Supporting Information

Figure S1 **Sequence of protospacer regions of** ***E. coli*** **CRISPR-4 spacers used to generate the WebLogo shown on** **Figure 1** **.** The protospacer sequences are underlined and mismatches with respect to the corresponding spacer are labeled in red. Nucleotides matching the PAM are bolded. Protospacer regions of LF82 spacers are marked with an asterisk.
(PDF)

Figure S2 **WebLogo generated by the alignment of 168** ***E. coli*** **CRISPR-4 spacers.**
(PDF)

Figure S3 **Schematic representation of the strategy used for synthesizing artificial CRISPR-4 arrays carrying a spacer identical to a P1 sequence.** The construction of the fragment carrying spacer P1.1 is shown to exemplify the general procedure. The leader of the CRISPR4.1 array, repeats and spacers are shown as green, black and blue boxes respectively. Relevant restriction sites as well as primers used for amplification of the leader-CRISPR region of the CRISPR4.1 of ED1a (C1.F and C2.R) and for synthesizing a fragment containing spacer P1.1 and a CRISPR unit (C3P1.F and C11.R) are indicated. Vertical lanes connecting the 3′ ends of primers C3P1.F and C11.R illustrate sequence complementarity at this region.
(TIF)

Table S1 **Plasmids constructed in this work carrying inserts with protospacer#1 and distinct PAM regions.**
(PDF)

Table S2 **Primers used for PCR reactions.**
(PDF)
