# Supplementary material for: Target Motifs Affecting Natural Immunity by a Constitutive CRISPR-Cas System in Escherichia coli
Source: PLoS One. 2012 Nov 26;7(11):e50797. doi: 10.1371/journal.pone.0050797 (PMC3506596; doi:10.1371/journal.pone.0050797)
Supplement: Figure S1 — Sequence of protospacer regions of E. coli CRISPR-4 spacers used to generate the WebLogo shown on Figure 1 . The protospacer sequences are underlined and mismatches with respect to the corresponding spacer are labeled in red. Nucleotides matching the PAM are bolded. Protospacer regions of LF82 spacers are marked with an asterisk. (PDF) [file pone.0050797.s001.pdf]

PAM

GATCTGAACAGCTATTGCATCAGCAGACCTGCAGCCACCTTCTTTCTGCGCGCCAGCGGTGAATCGATGAACCAGGCTGGCG
CTTCCAGCCACTGCGCGCCTTGTTCGGTCAGCGCAATCTGCTTCTTACCACCTTCCTCTTCGCGAATGGTAATCAGCGACTG
GCAATGGCTTCTGAGGCAGCGCCGGACGCCCTTTTGCCAGGATGTAAGCGCATTCGTGGCGATAGCCCACATATGCGGCCTT
TTCGGCGCTGAATATCACCGTCTTTGTTGACCAGTGTTTTAGTGACAAACGCTGCATGGTTTTTCGTAGTGTTGTATCCCTT
CATCCATATTATTTTTCTGAACAGTCTGTTTAACAGGACGAACTTCCAGGTATACCAGGTTATCTTCTTTATTAACCGCCCA
TGGCGGCTCGATCGTTCTGGAGATCGGGGAGCAAAAACACGCAAGGGATTTTGAGGCGGCAAAAGAGCAATGCAGGATAGAC
CGGAAGAAATACCGGATTCAGTCCGCTGGTCTTCCGGGATGTCCTCCAGGCGCGCCAGGACGGTTCTGATTTCATGATCGGA
CCTCGCCGTCAAAAAGACGACGGCTGCGGTATTCCACGGTCGGCCTGACCCGTTACCGGACGCGGTGAACAGCCCACAGGCA
GAAAAAAGATGCGCTGATGGTGAGCTTCTATGGCTGGAACCGCGTCGATCGCTTTATGGCCGCCTGGAAAAATGCGGGATTC
GCGCGAAAAACGCTTACAGAGGCTCTCAGGAGGTCAAGCGCGCCGATACCCCTAACCAGGGGCTTTCAGGTCGCCAGGAACC
TGTCGCATCCAGGATGAAACAACGTTGTCGCATCCTGGATGCGACAACACGCAACAAGGTGTTGCACAGATGTGTCATGATG
TTCAAGGTTTACACAGGTATCCCCAAAAGGTTGGGAACGTGTTCGAGAGCTGCTGAAGGATAGCCAAGGCATATCAGCACTG
GCTTTAACGGTGGTGTTCTGAACGGTGTTCCTTCTGCATATCACTGGTATACGGAACAGTATGGCGTGAAGTGGCCTTGTGG
CAGCCGTCAACTTCGTCACCCTCGACAACCTGTTTCGTTAGTCGCCGGAAATTTCCTGGTGCACTCGCGCCGCGAAAATCAA
GATATCGAACGCGATCTCGACAGGGTTGAATCCCTTGCGCGTGACGATACCGATGACGGGGGTTGAGTGGGCTGATAAATAT
CACAGCGGGAAACAGGCCGCAGAGGTGGCGGCAGGAGGGAGGACTGCTGCGCCATCAGGCGCAGTTTCCTCACACACCGTCA
ATCGTTGCCAAACAGGTAAGGCAGAAAGGAGAGACGGTCGCACTCTTCAACGTTCAGAGTGACAGGTTTCGCATATTGCATG*
CTGTTCTTCAGTGACTTAAGGGTAGGTACAAAGACGCCCAGACGCATCAGCGCCACAGGTTGTACGGTGTTGTTATTGTTTG*
CATCTTTGCTTTACGGTTTTTCATCTTACTGCGTACCCTTTCTTCCGCCTGTTCTGTGACGCGCTGGGCTTTTTGCAACAAC*
ATTGCTAAGGCAATGAATGAGGCAGACTTAGTTGTGGATGTCAGTACCACCTTTGAGGTGCCGAGGACTCTAGCGTTAAAAG
AACCCCAGACCCCCATCGCTGCAACACCGACGGTCTACGACCACCTACCAACCGCATGGCTGCCTGTTCCGTGCAAATGGGC
ACCGAGGATCTCGAAGAAGCAAAGAAAAATTCAAGGTTTACACAGGTATCCCCAAAAGGTTGGGAACGTGTTCGAGAGCTGC
TCACTTTGTGGGCTGAGCCAGGAGACATCAACATCCACTTCATTTGCTGAATACAGCGGCGTGCCATCCGTACCACAAAAGC
GTCGGCAAGTGCATACATAATGCGGGCGTTCGCAAAGGTTTTACCACACCCCGTCGAGGCCATATTGACGCCGAAAAATCCC
ACTATGTTCTAGCTCCGCCAGTTTGTTGGCATTATTGGCAGACAGGATCCGCGGCAAGGCGTTAATCGTCCGGTAGTCCGCG
CTGGAAAAATGCCGGGTACAGGTGAATGGTGGGCGGGTGGAGTATGTTACCAGCGAAGGTAAAGAGTCGTACTACTGGAATA
TGCGGAAGTGCTTCGCAAGAGTCGCCGGCAGAGGAGCAACAGTTCGTTAAGTACGAAGGCAGCCTGCATACCGGGGCGCTGG
CTTTAGTCACCCAATTGTCCAGTAAGGTATAAAAGTCGAGTTTTTCTTTCGGTTGACGGCAGGCCACACCGCGACTGGTTAC
CAGTCAGCATTAAGTTGCTTCTCCAGCCAACTTTCACACCCTTTACATGATTGTTCCGACTGTGGAGAAAGATTCTCCGGCA
ACCGCATGGCTGCCTGTTCCGTGCAAATGGGCTTTGCAGGGGGCTGGCGCGGTTCCTGGCGACCTGAAAGCCCCTGGTTAGG
CGTGAAATTAAATGGCGGATTATATATATCAGGCTCGCCAATTGTTAGTTTAAACGTGGAGATAGACCCAAATGGCTGAAAA
ATCGAAGCCAATACGACCGCGGATAAACATGATGTGATCGGCGTCTTCCGGCCACCACGTTTCACTTGTTGCCGCTTTAACG
TCCGGGACGCCAATCCCGACGCCAGATTTTGCTGGCGTACTGGGAATATAGCCCCGGATAACTGTTTGTGTCAATTAAGTGC
AGGGTAACGGGCCACGCTGGCGCGTCGCCCGTTACCCTTCCCGTTTGTGATGTGTCCGGAATGTTTTTTCCTGATGTTATGT
CTCAATTTTCAAATAGCCCGTTCAGTTCGCTGCAACCATTAGCCAAAACGGTAGGCTGGCTGATCCTTTCCCATCATCGAAT
GAGGAACACGTAACGCCCACCCTTTTCACGCATCGCCATTGTGTGATCCATGATGTGGGTCATGCCGGGATCGCCTGCTTCT
