# Supplementary figures and images for: Target Motifs Affecting Natural Immunity by a Constitutive CRISPR-Cas System in Escherichia coli
Source: PLoS One. 2012 Nov 26;7(11):e50797. doi: 10.1371/journal.pone.0050797 (PMC3506596; doi:10.1371/journal.pone.0050797)

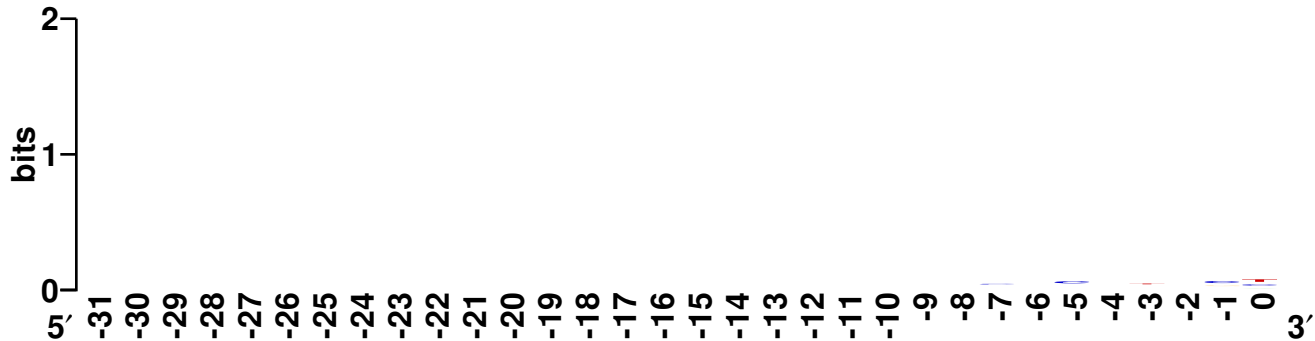

Supplement: Figure S2 — WebLogo generated by the alignment of 168 E. coli CRISPR-4 spacers. (PDF) [file pone.0050797.s002.pdf]

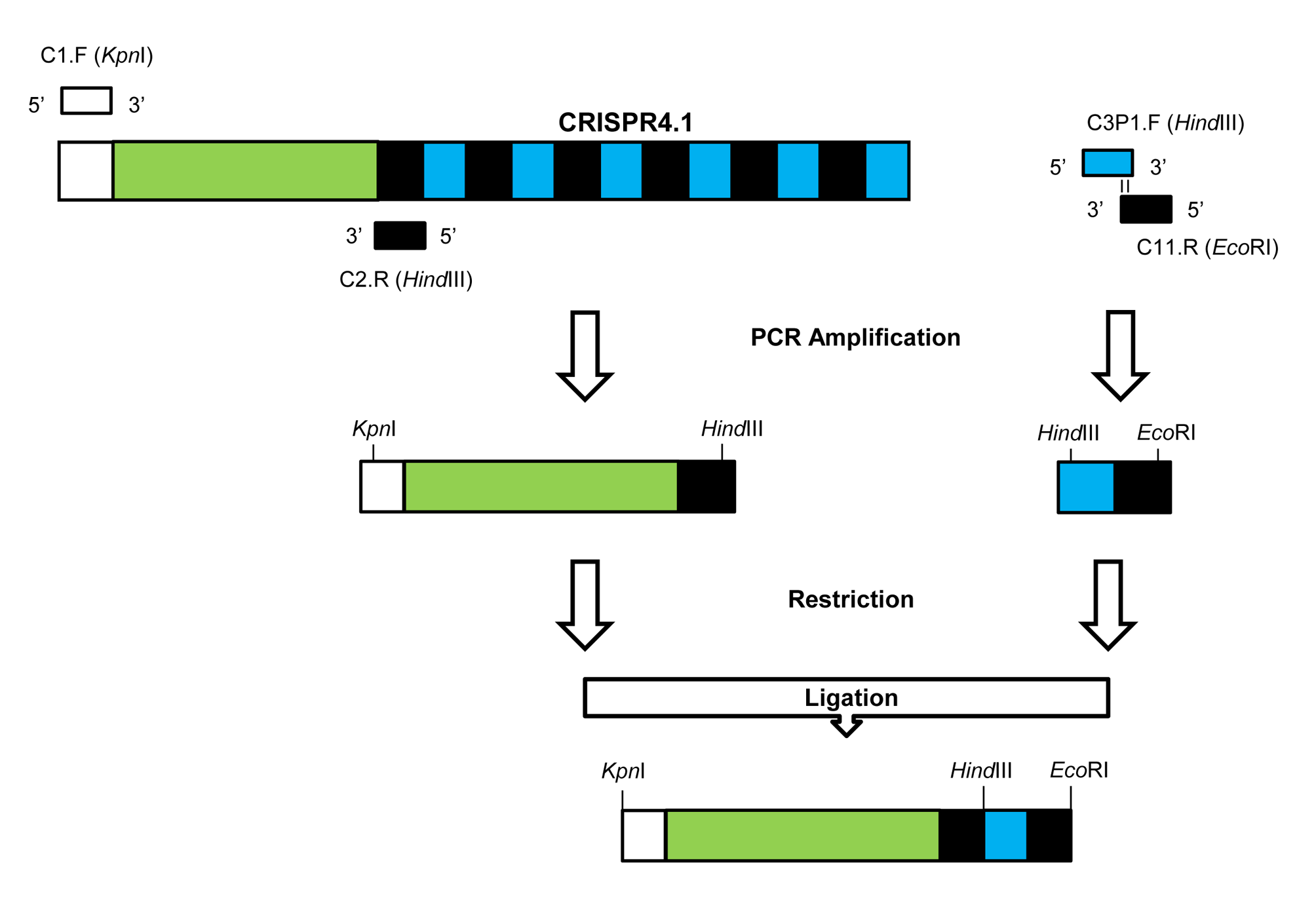

Supplement: Figure S3 — Schematic representation of the strategy used for synthesizing artificial CRISPR-4 arrays carrying a spacer identical to a P1 sequence. The construction of the fragment carrying spacer P1.1 is shown to exemplify the general procedure. The leader of the CRISPR4.1 array, repeats and spacers are shown as green, black and blue boxes respectively. Relevant restriction sites as well as primers used for amplification of the leader-CRISPR region of the CRISPR4.1 of ED1a (C1.F and C2.R) and for synthesizing a fragment containing spacer P1.1 and a CRISPR unit (C3P1.F and C11.R) are indicated. Vertical lanes connecting the 3′ ends of primers C3P1.F and C11.R illustrate sequence complementarity at this region. (TIF) [file pone.0050797.s003.tif]
