# Supplementary material for: Target Motifs Affecting Natural Immunity by a Constitutive CRISPR-Cas System in Escherichia coli
Source: PLoS One. 2012 Nov 26;7(11):e50797. doi: 10.1371/journal.pone.0050797 (PMC3506596; doi:10.1371/journal.pone.0050797)
Supplement: Table S1 — Plasmids constructed in this work carrying inserts with protospacer#1 and distinct PAM regions. (PDF) [file pone.0050797.s004.pdf]

Table S1. Plasmids constructed in this work carrying inserts with protospacer #1 and distinct PAM regions.

Plasmid	PAM region
**pCAR-GGC**	GGC
**pCAR-AGC**	AGC
**pCAR-AAC**	AAC
**pCAR-ATT**	ATT
**pCAR-GGG**	GGG
**pCAR-CCC**	CCC
**pCAR-GTT**	GTT
**pCAR-TGT**	TGT
**pCAR-TTG**	TTG
**pCAR-GAC**	GAC
**pCAR-TTT**	TTT
**pCAR-TGC**	TGC
**pCAR-AAG**	AAG
