# Supplementary material for: Target Motifs Affecting Natural Immunity by a Constitutive CRISPR-Cas System in Escherichia coli
Source: PLoS One. 2012 Nov 26;7(11):e50797. doi: 10.1371/journal.pone.0050797 (PMC3506596; doi:10.1371/journal.pone.0050797)
Supplement: Table S2 — Primers used for PCR reactions. (PDF) [file pone.0050797.s005.pdf]

Table S2. Primers used for PCR reactions.

Name	Sequence[a]	Purpose
**PAM.F**	GCCAGCAGCTTTCCAGCGAGCG	Synthesis of protospacer #1 - GGC[a]
**PAM23.F**	GCTAGCAGCTTTCCAGCGAGCG	Synthesis of protospacer #1 - AGC[a]
**PAM3.F**	GTTAGCAGCTTTCCAGCGAGCG	Synthesis of protospacer #1 - AAC[a]
**CR23.F**	AATAGCAGCTTTCCAGCGAGCG	Synthesis of protospacer #1 - AAT[a]
**3G.F**	CCCAGCAGCTTTCCAGCGAGCG	Synthesis of protospacer #1 - GGG[a]
**3C.F**	GGGAGCAGCTTTCCAGCGAGCG	Synthesis of protospacer #1 - CCC[a]
**LAG.F**	AACAGCAGCTTTCCAGCGAGCG	Synthesis of protospacer #1 - GTT[a]
**LA2G.F**	ACAAGCAGCTTTCCAGCGAGCG	Synthesis of protospacer #1 - TGT[a]
**LA3G.F**	CAAAGCAGCTTTCCAGCGAGCG	Synthesis of protospacer #1 - TTG[a]
**PAM1.F**	TTCAGCAGCTTTCCAGCGAGCG	Synthesis of protospacer #1 - GAC[a]
**LA4G.F**	CAAAAGCAGCTTTCCAGCGAGCG	Synthesis of protospacer #1 - TTT[a]
**PAM23T.F**	GCAAGCAGCTTTCCAGCGAGCG	Synthesis of protospacer #1 - TGC[a]
**2SPA4.1.F**	CTTAGCAGCTTTCCAGCGAGCG	Synthesis of protospacer #1 - AAG[a]
**SPA4.1_B.R**	GAGTGAGTTAACCGCGCTCG	Amplification of protospacer #1[a]
**RTCAS1.F**	GGTACAGGTGAATGGT	Amplification of *cas1*[b]
**RTCAS1.R**	TCGTTTGTCTTCAACA	Amplification of *cas1*[b]
**RTCSY1.F**	TCAGTCATGGTGATTCT	Amplification of *csy1*[b]
**RTCSY1.R**	GCAACAGGGAAATAGA	Amplification of *csy1*[b]
**RTTUFB.F**	TACTATCGGCCACGTT	Amplification of *tufB*[b]
**RTTUFB.R**	GATGATACCGCGTTCTA	Amplification of *tufB*[b]

[a] Cloning experiments.

[b] RT-PCR experiments.
